# Rhabdomyolysis in a patient with end-stage renal disease and SARS-CoV-2 infection: A case report

**DOI:** 10.1097/MD.0000000000036360

**Published:** 2023-12-01

**Authors:** Wenhui Lu, Xiaoying Li, Wenyi You, Rui Gong

**Affiliations:** a Department of Nephrology and Oncology, The People’s Hospital of Yubei District of Chongqing, Chongqing, China.

**Keywords:** COVID-19, end-stage renal disease, hemodialysis, rhabdomyolysis

## Abstract

**Rationale:**

Rhabdomyolysis can be an uncommon complication of coronavirus disease 2019 (COVID-19) infection. However, the diagnosis of rhabdomyolysis could be easily missed due to its atypical clinical presentations. We present a patient with a history of end-stage renal disease (ESRD) who contracted COVID-19 and subsequently developed rhabdomyolysis. We discuss and share our experience in the management of this patient.

**Patient concerns:**

An 85-year-old male with ESRD undergoing routine hemodialysis was tested positive for COVID-19. The patient had clinical symptoms of fatigue, muscle pain, and difficulty walking.

**Diagnosis:**

The serum creatine kinase (CK) level was markedly elevated to 32,492.9U/L, supporting the diagnosis of rhabdomyolysis. A computed tomography scan revealed muscle injuries throughout the body, confirming the diagnosis.

**Interventions:**

The patient was managed through electrolyte corrections and continuous renal replacement therapy.

**Outcomes:**

Repeat tests showed decreased levels of serum CK and negative severe acute respiratory syndrome coronavirus 2. His clinical symptoms, including fatigue and muscle pain, had significantly improved.

**Lessons:**

COVID-19 infection can cause muscle pain and fatigue, which can mask the symptoms of rhabdomyolysis. A missed diagnosis of rhabdomyolysis can be severe, especially in patients with ESRD. The serum CK level should be tested with clinical suspicion. Appropriate management, including adequate hydration and electrolyte balance, should be provided. Continuous renal replacement therapy should be considered in affected patients with renal insufficiency.

## 1. Introduction

Rhabdomyolysis is characterized by the disruption of the striated muscle cell membrane, which releases myocyte constituents into the extracellular fluids and bloodstream. Affected patients may experience systemic symptoms, including fever, fatigue, nausea, vomiting, palpitations, and muscle-related symptoms such as myalgia and extremity swelling. If left untreated, rhabdomyolysis can result in organ damage, such as acute renal failure, liver injury, and diffuse intravascular coagulation.^[1]^ Various factors can contribute to the development of rhabdomyolysis. It has been documented that patients with coronavirus disease 2019 (COVID-19), caused by the infection from severe acute respiratory syndrome coronavirus 2 (SARS-CoV-2), could experience muscle damage and exhibit elevated levels of creatine kinase (CK) in the peripheral circulation, with severe cases progressing to rhabdomyolysis.^[2]^ In this report, we present a case of a patient with a history of end-stage renal disease (ESRD) who contracted COVID-19 and subsequently developed rhabdomyolysis. The patient achieved a full recovery following prompt and individualized treatment. We aimed to describe the patient clinical course and management to enhance the clinical care provided to patients with similar conditions.

## 2. Case presentation

On December 2, 2022, an 85-year-old male reported fatigue, muscle pain and weakness, lower back pain, and difficulty walking. However, he denied fever, cough, abdominal pain or bloating, and diarrhea. He tested positive by the SARS-CoV-2 nucleic acid amplification test and was admitted to the Chongqing Yubei District People Hospital in Chongqing, China. One year before admission, the patient was diagnosed with ESRD, anemia of chronic renal disease, systemic lupus erythematosus, and stage 2 hypertension. He was treated intermittently with prednisone and received hemodialysis through the right arm arteriovenous fistula 3 times a week at an outside clinic. His last dialysis was performed on the same day of hospital admission. His medication included amlodipine for hypertension. During the hospital admission, his vital signs were temperature, blood pressure, respiratory rate, and heart rate. Physical examination revealed decreased muscle strength in 4 extremities. The patient COVID-19 vaccination history was unknown.

**Figure 1.**
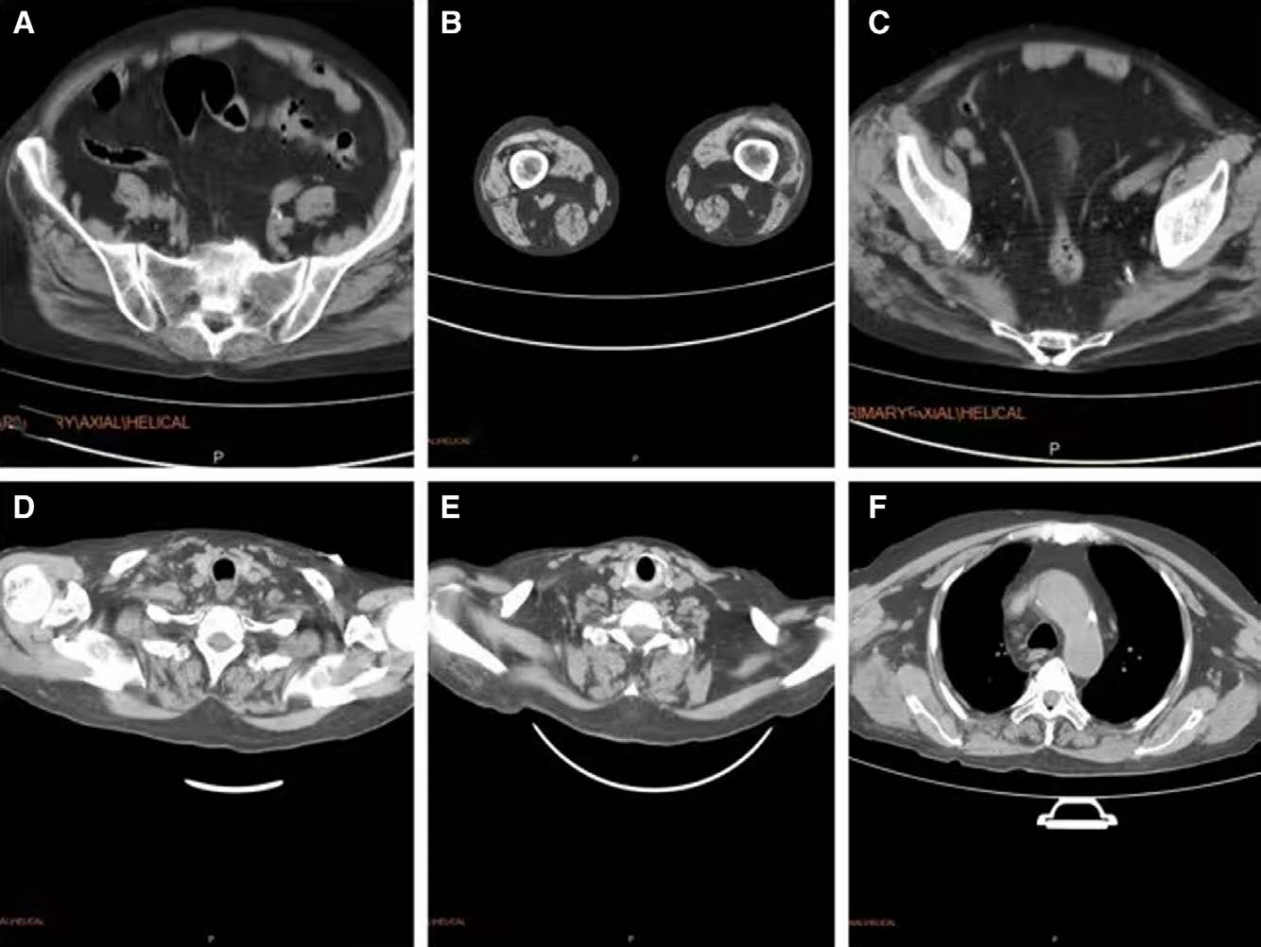
Computed tomography scan showed increased fat density shadows in the gluteus maximus, gluteus medius, and erector spinalis muscles (A), muscle atrophy, widened muscle spaces, and increased fat density shadows in the biceps femoris, intermediate femoris, and medial femoris muscles (B and C), increased fat density shadows in the levator scapula and trapezius muscles (D and E), and volume reduction, muscle atrophy, and increased fat density in the latissimus dorsi, scapula, pectoralis major, and pectoralis minor muscles (F).

**Figure 2.**
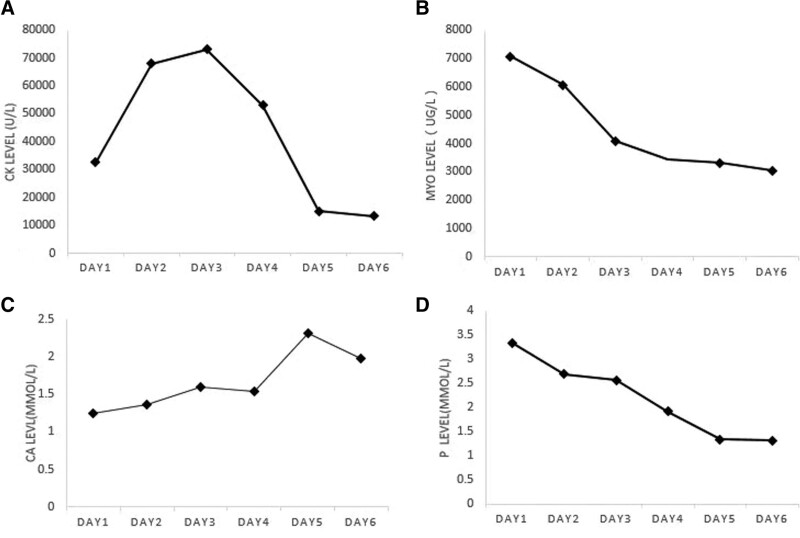
Serum creatine kinase (A), myoglobin (B), calcium (C), and phosphorus (D) levels.

On admission, the laboratory tests showed blood urea nitrogen 9.1 mmol/L, creatinine 520.8 µmol/L, uric acid 1160.7 µmol/L, CK 32,492.9 U/L (normal range 38–174 U/L), myoglobin 7065 U/L, lactate dehydrogenase 2041.2 U/L, and D-dimer 1.0 µg/mL. Blood chemistry results were potassium 5.4 mmol/L, calcium 1.3 mmol/L, and phosphorus 3.3 mmol/L. Hepatic function tests were alanine transaminase 31.8 U/L and aspartate aminotransferase 421.6 U/L. Blood gas analysis showed pH 7.33, PCO_2_ 31 mm Hg, SatO_2_ 99.3%, HCO_3_ 19.6 mmol/L, and normal autoimmune antibody spectrum. The computed tomography scan revealed blurred muscle textures in the bilateral latissimus dorsi and right upper limb, extensive atrophy of pelvic and lower limb muscles, widening muscle gaps, and increased fat density shadows. There were scattered inflammatory lesions in the middle and lower lobes of the right lung and the upper and lower lobes of the left lung (Fig. 1). The electrocardiogram indicated T-wave changes. The echocardiography showed an enlarged left atrium, mild pulmonary valve regurgitation, and decreased left ventricular diastolic compliance. Besides, continuous renal replacement therapy (CRRT) was performed with low molecular weight heparin anticoagulation on December 3, December 5, and December 7, 2022. In addition, the patient received intravenous calcium replacement. His clinical symptoms of fatigue and muscle pain had gradually improved. On December 7, reexamination was negative for the SARS-CoV-2. The renal function showed blood urea nitrogen 9.1 mmol/L, creatinine 896.7 µmol/L, uric acid 587.2 µmol/L, and D-dimer 0.9 µg/mL. The other tests included blood CK 13,204 U/L, myoglobin 3, 046 U/L, and lactate dehydrogenase 1458.02 U/L. Electrolytes were potassium 4.6 mmol/l, calcium 2.0 mmol/l, and phosphorus 1.3 mmol/l (Fig. 2). The patient also reported significantly improved fatigue and muscle pain. He was discharged and followed up in the clinic. His CK and myoglobin levels gradually return to normal.

## 3. Discussion

The SARS-CoV-2 pandemic has continuously received worldwide attention as numerous new characteristics of the virus infection are still being reported. SARS-CoV-2 causes multisystem disorders that damage various organs, such as the lungs, liver, kidneys, and gastrointestinal tract. Some patients could have persistent symptoms even months after recovery. Common symptoms include fever, cough, sore throat, fatigue, and loss of smell.^[3]^ In a study of 1099 COVID-19 patients in China, muscle pain happened in 14.9% of cases, with 0.2% of patients having rhabdomyolysis.^[4,5]^ The present case involves an elderly male with acute lower back pain, difficulty walking, and decreased muscle strength upon admission. These symptoms are often observed in COVID-19 patients. However, considering muscle pain could also indicate rhabdomyolysis, we tested the blood CK level >15,000 U/L. By most definitions, rhabdomyolysis is diagnosed if the CK level exceeds normal higher limits by more than 5 times or its absolute value surpasses 1000 U/L.^[6]^ Myoglobin levels also rose, further supporting the diagnosis of rhabdomyolysis.^[7]^ Only approximately 10% of patients with rhabdomyolysis have the typical triad of acute or subacute myalgia, transient myasthenia, and dark urine. As a result, nonspecific symptoms are also common, requiring special clinical attention when diagnosing this disorder. In this particular case, the patient had an underlying ESRD. His urine color could not be observed. We performed the blood test and made the diagnosis of rhabdomyolysis. The whole-body CT scan reported multiple muscle lysis sites, consistent with a diagnosis of rhabdomyolysis.

Rhabdomyolysis can have different etiologies, including trauma, drug toxicity, intense exercise, genetic metabolic diseases, endocrine disorders, metabolic abnormalities, infections, high fever, hypothermia, status epilepticus, and influenza.^[8]^ Identification of the etiology can guide its clinical management. Our patient denied a history of drug abuse, intense exercise, or trauma before the disease onset. Immunological tests also revealed no abnormalities, ruling out the possibility of genetic metabolic diseases. Several studies have reported that the novel coronavirus, a member of the Coronaviridae β coronavirus genus, was associated with acute renal failure and rhabdomyolysis.^[9–11]^ Evidence of myocardial injury was frequently observed in SARS-CoV-2 patients,^[12]^ indicating that muscle cell damage is a common and potential disease mechanism. In addition, recent studies have suggested that SARS-CoV-2 could cause extrapulmonary involvements by entering the small vessel endothelium, intestines, synovial tissue, and other organs through the angiotensin-converting enzyme 2 (ACE-2) receptor.^[13]^ Like these susceptible organs, muscle tissues also express the ACE-2 receptors, making them a target for the SARS-CoV-2 virus.^[14,15]^ The SARS-CoV-2 spike protein binds to the ACE-2 receptor, allowing the virus to infect muscle fibers and leading to muscle damage. However, other studies in COVID-19 patients with rhabdomyolysis revealed no viral particles in the muscles. This suggested that muscles were injured by the excessive immune responses but not by the direct SARS-CoV-2 infection.^[16]^

Rhabdomyolysis often leads to acute kidney injury, accounting for about 15% of all patients with acute kidney injury.^[17]^ It is commonly accompanied by severe electrolyte imbalances that can be life-threatening.^[18,19]^ Treatments of rhabdomyolysis primarily involve addressing the underlying cause, hydration, correcting electrolyte imbalance, and dialysis when necessary. Our patient had underlying ESRD and was already on dialysis treatment. His COVID-19 infection was treated with supportive care.

Patients with rhabdomyolysis commonly experience life-threatening complications, including hyperkalemia, hypercalcemia, hyperazotemia, and hyperuricemia, especially in patients with acute renal function damage.^[20]^ Extensive muscle cell damage can cause a large influx of plasma calcium into the damaged muscles, leading to calcium phosphate precipitation and inhibition of calcitriol and parathyroid hormone resistance due to hyperphosphatemia. As a result, patients with rhabdomyolysis often present with hypocalcemia as an early manifestation. In addition, releasing inorganic and organic phosphorus components can lead to hyperphosphatemia. The initial calcium encapsulated within muscle cell cytoplasm is released back into the plasma following complete cell necrosis, leading to hypercalcemia in patients with rhabdomyolysis. On the second day of admission, the blood calcium level in our patient reached its lowest point (1.3 mmol/L). Additionally, blood phosphorus reached its highest level on admission at 3.9 mmol/L. Therefore, we administered calcium supplementation to this patient during the early stages of treatment. Early intensive rehydration therapy is the cornerstone of rhabdomyolysis management. However, due to the patient ESRD, we only gave him limited intravenous hydration but performed CRRT. Previous clinical studies have demonstrated that renal replacement treatment should not only rely on eliminating serum concentrations of myoglobin or CK but also consider the extent of renal damage and the management of associated complications.^[21]^

CRRT, instead of conventional dialysis, was administered during treatment. There were several reasons for choosing CRRT for this patient. Firstly, the patient exhibited a high level of myoglobin released from the damaged muscle cells. Myoglobin is a large molecule with a molecular weight of 17.6 kDa. CRRT is more effective than conventional dialysis in clearing medium and large molecules from the bloodstream.^[22]^ Secondly, CRRT allows for dynamic adjustment of electrolyte and pH concentrations in the replacement solution based on real-time blood gas analysis results, providing superior control compared to conventional dialysis.^[23]^ Following CRRT treatment, the patient experienced alleviation of clinical symptoms and decreased serum levels of CK and myoglobin. Furthermore, the patient hypocalcemia, hyperkalemia, and hyperphosphatemia were corrected, indicating the effectiveness of CRRT in addressing these electrolyte imbalances.

The limitation of our study was that we had a single case report. We did not know the actual incidence of rhabdomyolysis in COVID-19 patients. We also did not know if renal failure and ESRD could increase the risk of rhabdomyolysis in these patients. Future studies with a large sample size can be performed to address these questions.

In conclusion, it is essential to recognize that COVID-19 patients are at a high risk of developing rhabdomyolysis. Diagnosing rhabdomyolysis in these patients can be challenging due to overlapping clinical symptoms such as muscle pain and weakness associated with COVID-19 infection. Therefore, it is crucial to test the serum CK level in patients with strong clinical suspicion. The mainstays of treatment for rhabdomyolysis include addressing the underlying causes, adequate hydration, and correction of electrolyte imbalances.

## Author contributions

**Conceptualization:** Rui Gong.

**Data curation:** Wenhui Lu, Xiaoying Li.

**Formal analysis:** Wenhui Lu, Xiaoying Li.

**Project administration:** Rui Gong.

**Writing – original draft:** Wenhui Lu, Xiaoying Li.

**Writing – review & editing:** Wenyi You.
